# Linolenic Acid-Metronidazole: a Compound Relieving Drug Resistance and Inhibiting Helicobacter pylori

**DOI:** 10.1128/aac.00073-22

**Published:** 2022-06-27

**Authors:** Yuan-Yuan Dai, Chun Qin, Gan-Rong Huang, Yan-Chun Qin, Yong-Yi Huang, Yan-Qiang Huang, Li-juan Zhao

**Affiliations:** a Research Center for the Prevention and Treatment of Drug Resistant Microbial Infecting, Youjiang Medical University for Nationalities, Baise, Guangxi Zhuang Autonomous Region, China

**Keywords:** *Helicobacter pylori*, metronidazole, inolenic acid, Lla-Met, Met resistance

## Abstract

Metronidazole (Met) is the first choice for treating Helicobacter pylori (*Hp*). However, *Hp* is easy to resistant, making Met unable to be widely used. How to overcome *Hp*’s Met resistance is still an issue. In this study, Met was used as the primary raw material with linolenic acid to prepare a novel compound-linolenic acid-metronidazole (Lla-Met). The MIC, minimum bactericidal concentration (MBC), colonization amount of *Hp* in gastric mucosa, etc., were evaluated, respectively. Lla-Met was successfully prepared by the detection of nuclear magnetic resonance, etc., and its MIC and MBC to *Hp* were 2~4 μg/mL, 8~16 μg/mL. Moreover, *in vivo* experiments, Lla-Met significantly reduced the colonization of drug-resistant *Hp* in gastric mucosa. In the toxicity test, Lla-Met inhibited rate to GES-1 and BGC823 cells were 15% at 128 μg/mL; the mice were administered 10 times treatment Lla-Met treatment (240 mg/kg), have no difference significant injuries were found in their stomach, liver, spleen, kidney, and weight. In addition, *Hp* G27 continued for 18 days *in vitro* with sub-Lla-Met concentration, G27 did not show drug resistance to Lla-Met; Lla-Met did not exert an effect on non-*Hp* species with 128 μg/mL; Compared with a neutral environment, when the acid concentration is 3.0, Lla-Met is not decomposed and has better stability. Conclusion: Lla-Met, a newly prepared compound, has relatively well antibacterial of Met-resistant and sensitive *Hp*, with a capability of overcoming the metronidazole resistance of *Hp.*

## INTRODUCTION

Helicobacter pylori (*Hp*), a Gram-negative microaerophilic pathogen that colonizes the epithelial cells of the gastric mucosa (1), infects more than half of the world’s population (2, 3), the infection rate in some underdeveloped countries exceeding 90% (4, 5). The infection of *Hp* can lead to the diseases in the upper digestive system, such as chronic gastritis, peptic ulcer, gastric cancer, and mucosa-associated lymphoid tissue (*MALT*) lymphoma (3, 6–8), while being closely associated with a variety of diseases outside the gastrointestinal tract (9, 10).

*Hp* is classified as a Class I carcinogen (11, 12). *Hp* infection is a contagious disease that can be transmitted through fecal-oral transmission (9, 13). The eradication of *Hp* can promote the healing of patients with peptic ulcer and delay the occurrence and development of gastric cancer (14, 15). Besides, *MALT* lymphoma, which is mainly caused by *Hp*, is the first cancer cured by antibiotic treatment (16, 17).

The eradication of *Hp* is regarded as an important means of preventing the occurrence and development of digestive system diseases. However, the standard triple therapy (PPI + amoxicillin + metronidazole/clarithromycin) remains the treatment of choice for *Hp* in regions and countries where the clarithromycin resistance rate is less than 15% (13). Nevertheless, with the long-term use of this therapy, *Hp*’s resistance has continued to increase (18), and its high resistance to metronidazole and levofloxacin is the main reason for the failure of eradication (19, 20). For instance, the research in the Asia-Pacific region (21) found that *Hp*’s metronidazole resistance rates over 40% in most regions and in some underdeveloped regions even exceeds 80%. Meanwhile, *Hp* has been listed as a priority for antibiotic resistance by the World Health Organization in 2017 (22).

In addition to drug resistance, there are also some challenges faced by the standard triple therapy, including the premature destruction of antibiotics due to the acidic environment in the stomach and the low blood concentration of drug reaching the gastric mucosa, which cannot eradicate *Hp* effectively; combined antibiotics can disturb the intestinal flora and a host of problems. There is therefore an urgent demand for a new targeted and highly acid-resistant agent to prevent drug resistance, especially *Hp* resistance to metronidazole and eradicate *Hp* infection.

There are many ways to realize new *Hp* antimicrobial agents, such as screening monomer compositions with antibacterial activity from the existing compound libraries and effective monomer compositions from Chinese herbal medicine compound preparations and modifying the structure of the existing antibacterial drugs, et al. Metronidazole has low cost, good antibacterial effect, and few side effects, and is used to treat anaerobic infections, while due to *Hp*’s high drug resistance, it is rarely used now as first-line therapy. Therefore, how to overcome the resistance of *Hp*’s metronidazole is a difficult problem to restore its first-line treatment.

Previous research has proved that unsaturated fatty acids and their derivative liposomes are ideal antibacterial agents and show good antibacterial activity against a variety of bacteria (23, 24). The unsaturated fatty acids have a certain antibacterial effect on *Hp* and linolenic acid liposomes help to remove *Hp* in the gastric mucosa of mice and reduce the inflammatory response in their stomachs (25). Besides, Petschow (26) proved that unsaturated fatty acids have an antibacterial effect to different strengths against *Hp*, which is affected by factors such as the length of the carbon chain and the position of the double bond. Unsaturated fatty acids, including lauric acid, sub-acid, linolenic acid, etc., were found to barely develop *Hp*’s resistance. Among which linolenic acid, an essential unsaturated fatty acid that cannot be synthesized in the body, is widely found in plants such as flax, soybeans, rapeseed, etc. (27). With its ability to lower blood lipid levels and blood pressure, preventing cardiovascular and cerebrovascular diseases, fighting inflammation, etc. (28, 29), linolenic acid is an ideal natural antibacterial agent, Lee et al. reported that the MIC of linolenic acid to Staphylococcus aureus and Bacillus cereus were 10 and 50 ppm, respectively (30). Furthermore, linolenic acid is unstable in nature, can be chemically combined with a variety of substances and prevent *Hp* developing from resistance (31), can kill resistant *Hp* and spherical Hp (31), making it stand out among many unsaturated fatty acids. However, there have been many reports on making *Hp* preparations by using linolenic acid as raw materials with a better antibacterial effect against *Hp*. For example, Huang (32) reacted linolenic acid with metallic zinc to prepare zinc linoleate, a compound with its MIC to sensitive and drug-resistant *Hp* strains reaching 2~8 μg/mL. Obonyo (31) prepared linolenic acid into a nano-preparation, whose antibacterial mechanism is mainly to destroy the bacterial cell membrane with a good antibacterial effect against the sensitive and drug-resistant *Hp* strains.

Despite all the aforementioned methods, the chemical combination of linolenic acid and antibiotics has not yet been reported. To overcome the *Hp*’s high metronidazole resistance, we prepared Lla-Met compound by esterification of linolenic acid with metronidazole. Meanwhile, evaluated the inhibiting effects of this compound on sensitive and resistant *Hp* strains *in vivo* and *in vitro*, and evaluated the specificity and stability, in the hope of restoring the sensitivity of clinically drug-resistant *Hp* strains to metronidazole and providing a new candidate drug for clinical treatment of *Hp*.

## RESULTS

### Synthesis and characterization of Lla-Met.

**FIG 1:**
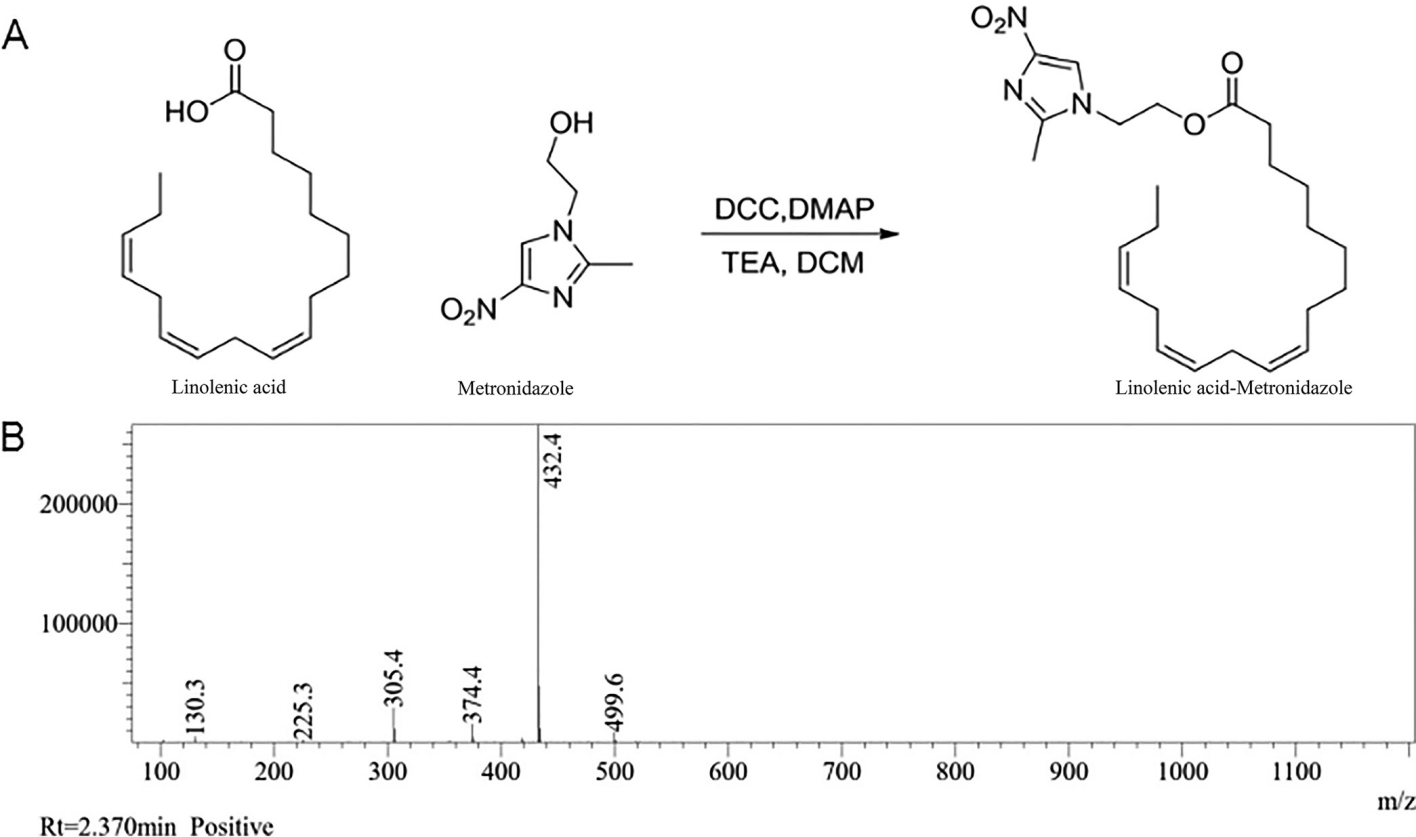
Lla-Met synthesis route and characterization results. (A) The synthesis route of the Lla-Met; (B) the mass spectrometry identification results of Lla-Met. The molecular formula after hydrogenation was 432.28 (similar to 432.4 found elsewhere).

**FIG 2:**
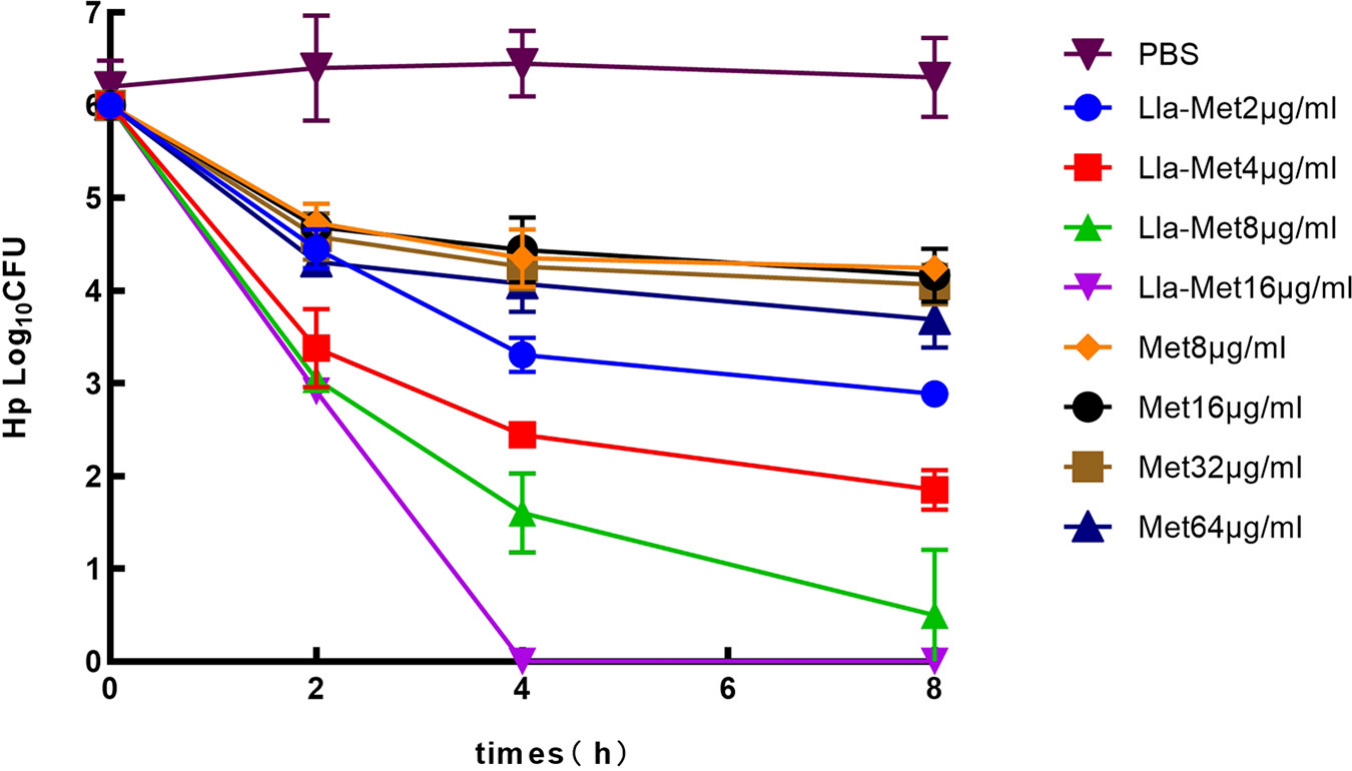
Illustrates the MBC of different multiples of Lla-Met for the drug-resistant *Hp*159. Lla-Met had a bactericidal activity against 159 in a concentration-time-dependent manner. When 159 was exposed to 4 μg/mL and 8 μg/mL linolenic acid-metronidazole, the MBC could be reached after 3 h and 2 h; metronidazole expoured at 32 μg/mL did not reach MBC within 8 h, indicating that Lla-Met had better bactericidal activity against drug-resistant *Hp*159 compared to metronidazole at the same MIC multiplicity. PBS was used as a negative control.

**FIG 3:**
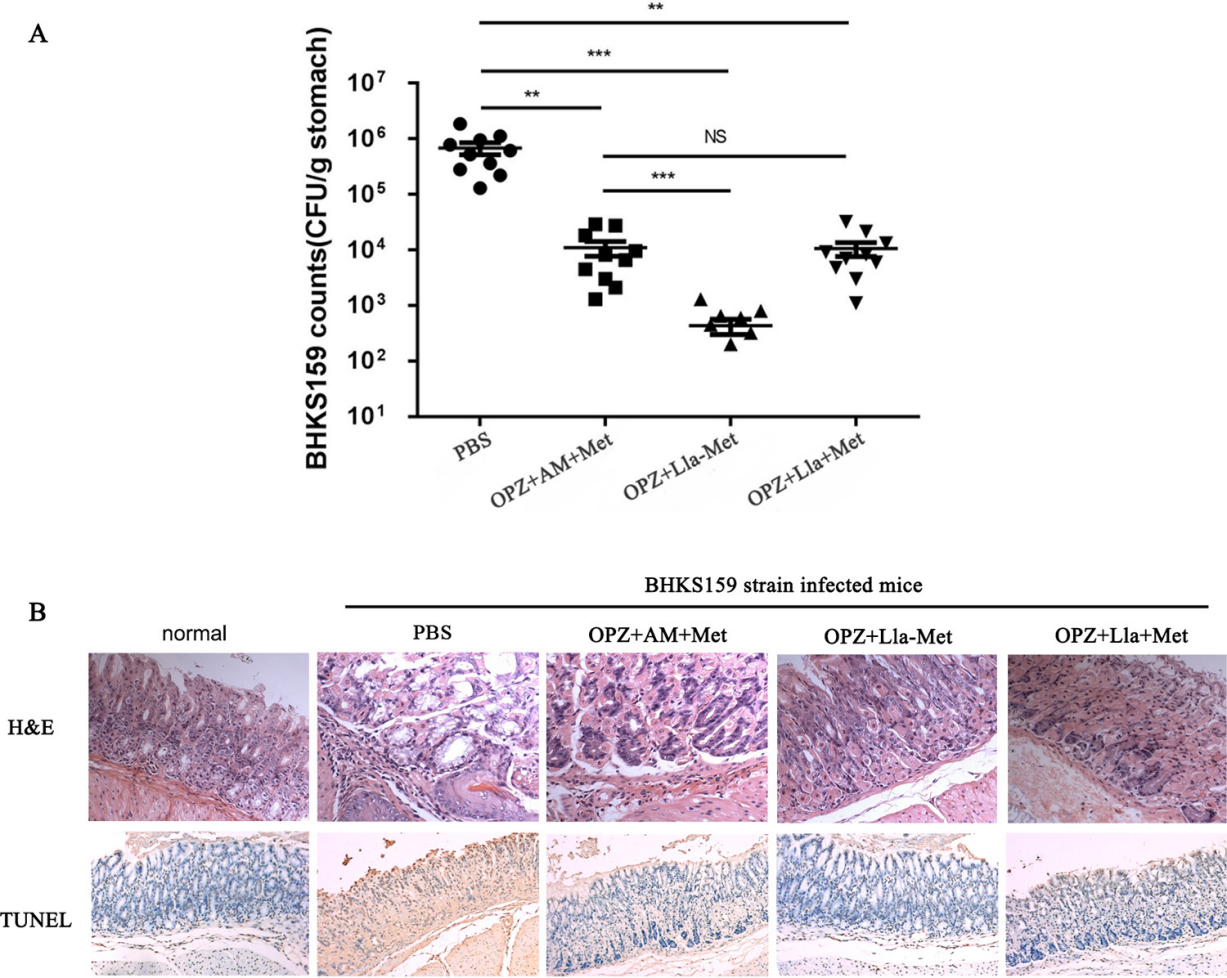
Efficacy evaluation of Lla-Met *in vivo*. (A) The amount of *Hp* colonization in the gastric mucosae of mice after Lla-Met treatment. PBS was used as the negative control and OPZ+AM +Met refers to omeprazole+amoxicillin+metronidazole (standard triple); OPZ+Lla-Met refers to the omeprazole+Linolenic acid-Metronidazole compound; OPZ+Lla+Met refers to the omeprazole+linolenic acid+metronidazole; among them, OPZ+Lla-Met had better efficacy than the standard triple therapy (*P* < 0.05), with the difference statistically significant; (B) HE and TUNEL staining of gastric mucosal tissues of mice. In the OPZ+Lla-Met group, a clear structure of the gastric mucosal tissues with less inflammatory cell infiltration demonstrated that Lla-Met could repair the inflammatory response in the stomach.

Linolenic acid can undergo an esterification reaction with metronidazole in the presence of DCM, DCC, DMAP, and diethylamine (Fig. 1A). After the reaction solution was purified, the synthesized product was identified as Lla-Met using ^1H-NMR^, ^13^C-NMR, mass spectrometry, and infrared spectroscopy. Using mass spectrometry, the characteristic absorption peak of Lla-Met compound was found at 432.4 nm, with its chemical formula posited to be C_24_H_37_N_3_O_4_[M+H]^+^ and a molecular weight of 431.28, a figure that was similar to that after hydrogenation (432.28, Fig. 1B). Besides, Lla-Met was also identified by ^13^C-NMR, ^1H-NMR^, and infrared spectroscopy with supplemental material shown in Fig. 1 to 3.

### The inhibitory effect of Lla-Met compound on *Hp*.

Table 1 displays the MICs of Lla-Met for *Hp* standard strains and Met-resistant strains. The MICs of Lla-Met were 2~4 μg/mL. For Met-resistant strains, the MIC of metronidazole was 16~64 μg/mL, linolenic acid was 128 μg/mL; a combination of linolenic acid and metronidazole was 8 μg/mL and 32 μg/mL. The antibacterial effect of Lla-Met was significantly better than those of linolenic acid alone, and linolenic acid in combination with metronidazole. The MIC of Lla-Met is 8 to 32 times lower than that of MET, and Lla-Met provided the significant antibacterial activity against drug-resistant *Hp*.

**TABLE 1:** The MIC of Lla-Met compound for *Hp* (μg/mL)^*a*^

Bacterial strain	Met-resistant strain	Linolenic acid	Metronidazole	Linolenic acid + metronidazole	Linolenic acid −metronidazole
HP26695	−	>128	4	8 + 8	4
HPG27	−	128	4	8 + 8	2
HP159	+	128	16	16 + 16	4
HPBS002	+	128	16	16 + 16	4
HPBS005	+	128	16	16 + 16	4
HPBS007	+	>128	16	16 + 16	2
HPBS011	+	>128	64	32 + 32	4
HPBS014	+	>128	64	32 + 32	4

*^a^*“+” refers to Met resistant strains; “−” refers to Met sensitive strains. Metronidazole: ≤8 μg/mL for susceptible; ≥8 μg/mL for resistant. The MIC of Lla-Met for sensitive and drug-resistant of *Hp* strains were 2–4 μg/mL; metronidazole and PBS were used as controls; the MIC of linolenic acid + metronidazole were 32 μg/mL, and 32 μg/mL respectively, expressed as the “32 + 32”; the MIC was the lowest drug concentration that inhibited 90% bacterial growth. In case of a single jumping hole, the highest drug concentration that inhibits bacterial growth should be recorded; if multiple jumping holes occur, the results of this experiment are invalidated.

Fig. 2 shows the time-kill dependence curve of different concentrations of linolenic acid-metronidazole on resistant *Hp*159. As the concentration of Lla-Met increased, the time required for *Hp* to reach the MBC decreased in a dose-and time-dependent manner. However, metronidazole had not yet reached the minimum bactericidal curve and there was no significant time-concentration dependence, indicating Lla-Met was significantly more effective against drug-resistant *Hp*159.

### The inhibitory effect of Lla-Met compound on non-*Hp*.

Table 2 lists the antibacterial spectrum detection results of Lla-Met against 20 strains of non-*Hp*. The MICs of Lla-Met were found to be all greater than 128 μg/mL, suggesting that Lla-Met provided specific antibacterial activity against *Hp* without causing an imbalance of the flora outside the intestine.

**TABLE 2:** MIC of Lla-Met compound for non-*Hp* (μg/mL)

Bacterial strain	MIC of Lla-Met (μg/mL)
Staphylococcus aureus	>128
Escherichia coli	>128
Pseudomonas aeruginosa	>128
Acinetobacter baumannii	>128
Klebsiella pneumoniae	>128
Candida albicans	>128
Cryptococcus neoformans	>128
Saccharomyces cerevisiae	>128
Bacillus subtilis	>128
Stenotrophomonas maltophilia	>128
Lactobacillus acidophilus	>128
Acetobacter pasteurianus	>128
Candida tropicalis	>128
Lactobacillus curvatus	>128
Morganella morganii	>128
Campylobacter jejuni	>128
Bacteroides fragilis	>128
Bifidobacterium longum	>128
Enterobacter hormaechei	>128
Staphylococcus haemolyticus	>128

### Efficacy evaluation of Lla-Met compound *in vivo*.

The results of the animal experiments *in vivo* showed that the amount of *Hp* colonization in the gastric mucosae of mice was significantly reduced after treatment with OPZ+ Lla-Met, and the inhibitory effect was significantly better than that of standard triplet and OPZ+ Lla +Met (*P < *0.05). The HE and TUNEL stainings of mice gastric mucosal tissue showed that the degree of inflammatory cells and inflammatory factors infiltration in the OPZ+ Lla-Met treatment group was significantly lower than that the standard triple, OPZ+ Lla +Met; implying that OPZ+ Lla-Me could not only significantly reduce the amount of *Hp* colonization in the gastric mucosae, but alleviate the inflammatory response in the stomach of mice (Fig. 3A and B).

### Safety evaluation of Lla-Met compound.

**FIG 4:**
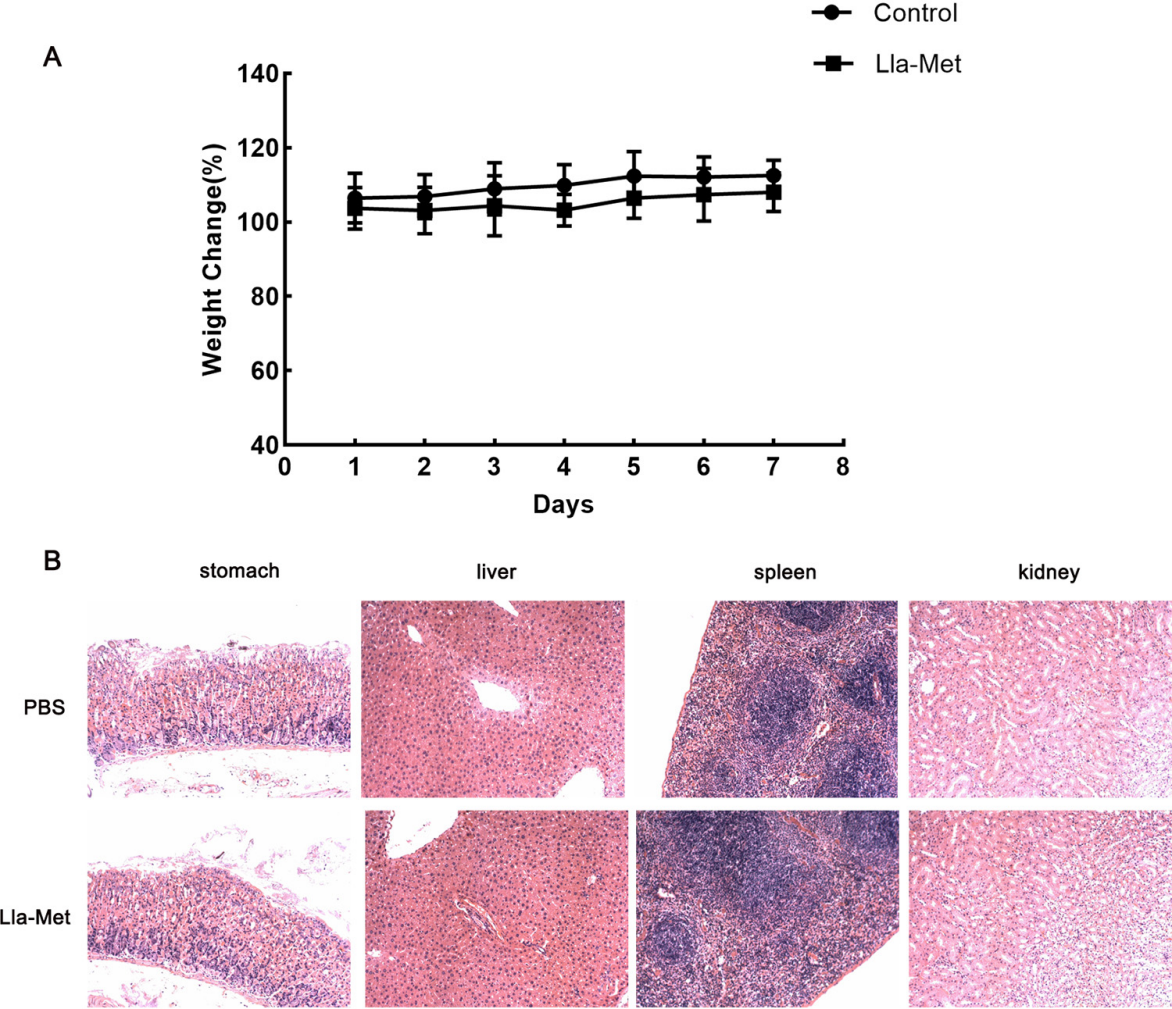
Safety evaluation of Lla-Met *in vivo*. A. Mice were treated with 10 times treatment dose of Lla-Met (240 mg/kg). B. The stomach, liver, spleen, and kidney of mice not were injured with 10 times treatment dose of Lla-Met by H&E.

**FIG 5:**
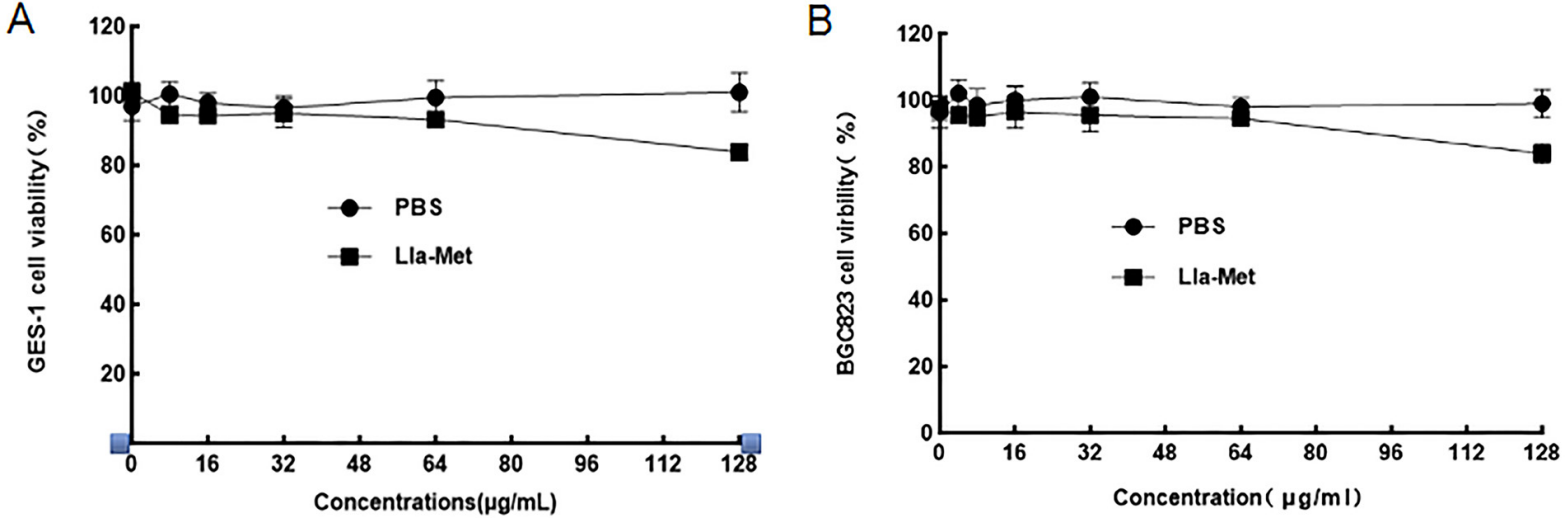
A and B displayed Lla-Met effects on the viability of BGC803 and GES-1 cells; as the concentration of Lla-Met increased, there was no significant change in cell viability compared with the PBS group. The cytotoxicity of Lla-Met at 128 μg/mL (128 times of MIC) concentration is still very lower. It shows that Lla-Met has high biosafety *in vitro*. Three biological repeats were performed for each experiment.

The toxicity of Lla-Met was evaluated *in vivo* and *in vitro*. Changes in weights and liver, spleen and kidney tissues of mice after administration of 10 times the therapeutic dose of Lla-Met are presented as follows: after 7 days of large doses of Lla-Met continuous administration, the weights and visceral of the mice did not change significantly compared with those of the PBS group (Fig. 4A and B); The cytostatic rate of 128 μg/mL Lla-Met was about 15%, with no obvious toxicity to BGC823 and GES-1 cells compared with PBS (Fig. 5A and B).

### Evaluation of Lla-Met’s stability and *Hp*’s Lla-Met resistance.

**FIG 6:**
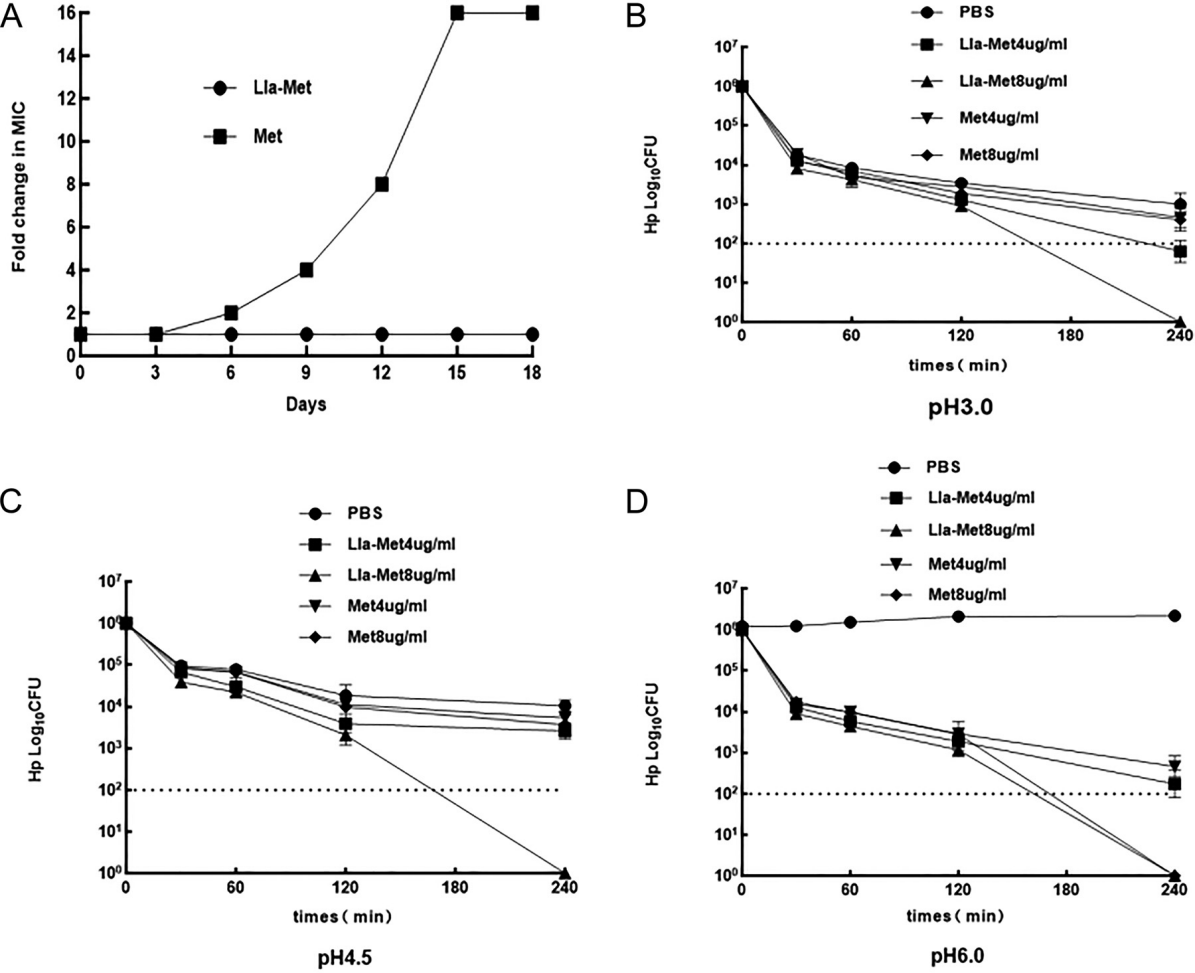
Resistanced induction of Lla-Met and Stability under acidic conditions. (A) *Hp* MIC has no change after 18 days of continuous induction in 0.25 μg/mL Lla-Met (1/4MIC) concentration during resistance induction, with 1 μg/mL Met (1/4MIC) concentration as the control; (B) the bactericidal activity of Lla-Met at pH 3.0; (C) the bactericidal activity of Lla-Met at pH 4.5; (D) the bactericidal activity of Lla-Met at pH 6.0; metronidazole and PBS were used as controls. The dotted line of B, C, and D represent the limit of detection of the bacteria. It stands for If 1 colony grew out, would that be about 100 bacterias.

To evaluate *Hp*’s drug resistance to Lla-Met, we examined the stability and bactericidal activity of Lla-Met under acidic conditions. Meanwhile, *Hp*G27 was continuously induced at Lla-Met 1/4 MIC of Lla-Met (0.25 μg/mL) concentration to observe whether *Hp* was prone to Lla-Met resistance. As a result, the MIC multiple of Lla-Met did not change when exposed to 0.25 μg/mL Lla-Met for serial passaging for 18 days; metronidazole was induced under the same conditions, with its MIC multiple increases 2-fold on the 6th day, and increases by 16 times on the 15th day compared with the first day. This result, however, noted that *Hp* was susceptible to resistance under condition of prolonged exposure to metronidazole, while it was less susceptible to Lla-Met, as shown in Fig. 6A. Meanwhile, the bactericidal activity of Lla-Met on G27 under acidic conditions of pH 3.0, 4.5, and 6.0 was more stable with the antibacterial effect more significant than that of metronidazole (Fig. 6B to D). In addition, Lla-Met was placed in pH 3.0 and pH 7.0 solutions at room temperature for a month, TLC was used to assess the stability of Lla-Met under strongly acidic conditions, founding point pH 7.0 and pH 3.0 chromatographic plates were on a horizontal line. The structure of Lla-Met did not change after it was placed at room temperature in the strong acid environment for 1 month, so it was not decomposed. This indicates that Lla-Met can stably exist in strongly acidic solutions (Fig. S4).

## DISCUSSION

As the resistance of *Hp* to antibiotics increasing year-on-year, the effectiveness of *Hp* treatment decreases accordingly (33, 34). Therefore, there is an urgent need for novel *Hp* preparations that can prevent *Hp* from developing drug resistance. Linolenic acid, which is an essential unsaturated fatty acid, is an ideal anti-*Hp* drug candidate due to its active chemical properties, broad-spectrum antibacterial effect, safely inhibiting development of drug resistance. However, studies on the synthesis of linolenic acid derivatives using linolenic acid as the raw materials, such as Zinc Linolenate and Linolenic acid liposome et al., which have a good antibacterial effect on *Hp* (31, 32). Metronidazole is a conventional drug for the treatment of anaerobic bacteria, however, the high drug resistance of *Hp* to metronidazole leads to the year-by-year decrease of clinical eradication rates of *Hp*. Therefore, in our study, linolenic acid and metronidazole were used as raw materials to prepare Lla-Met compound to give full play to their antibacterial advantages. Their antibacterial effects on *Hp* were also explored. Lla-Met was demonstrated a good antibacterial effect on sensitive *Hp* standard strains and Met-resistant *Hp* strains, the MIC was 2~4 μg/mL, antibacterial effect significantly better than the combined use of linolenic acid and metronidazole, and linolenic acid alone; Lla-Met could act specifically on *Hp* strains, without having antibacterial effects on other strains, as shown in the antibacterial spectrum. The MBC concentration of Lla-Met on *Hp*159 strains that were drug-resistant to metronidazole increased in a concentration and time-dependent manner, with the MBC level reached within 2~4h, indicating this compound was a more ideal an antibacterial agent compared with metronidazole which had not yet reached the MBC level at 8 h under the same MIC multiple. Besides, after 18 days of the selection, *Hp*’s resistance to Lla-Met compound, its MIC had no changed, while to metronidazole had increased by 16 times. This finding implied that *Hp* was less easy developing drug resistance to Lla-Met in long-term exposure. In addition, the acidic test results revealed, Lla-Met had more stable chemical properties under acidic conditions and a better antibacterial effect when it compared to metronidazole.

After the acute gastritis infection of mice was treated with Lla-Met, the amount of *Hp* colonization was significantly reduced compared with that of those treated with the standard triplet of omeprazole, amoxicillin and metronidazole; through HE staining of the gastric mucosa tissues of mice, showed less inflammatory cell infiltration compared to standard triplet and OPZ+Met+Lla, suggesting that Lla-Met could significantly improve the inflammatory response of acute gastritis in the stomachs of mice. *In vitro* cytotoxicity experiments showed that Lla-Met had a lower cytotoxicity to GES-1 and BGC823 cells, with the cell survival rate remaining as high as 86% at a concentration of 128 μg/mL (128 times MIC). On the other hand, *in vivo* toxicity experiments in mice showed that after the administration of high-dose Lla-Met, the weights, livers, spleens, and kidneys of the mice did not change significantly, indicating that Lla-Met exerted a better antibacterial effect *in vivo* and high biosafety.

In summary, Lla-Metcompound we prepared has a good antibacterial effect on Met-resistant *Hp* both *in vivo* and *in vitro*, a strong specificity, and high safety. Under acidic conditions, it is more stable and has a significantly better antibacterial effect and can overcome the shortcomings of metronidazole, such as easily making *Hp* with drug resistance, making it a new, clinically ideal, anti-*Hp* preparation.

## MATERIALS AND METHODS

### Materials and equipment.

Linolenic acid (batch number: 463-40-1) was purchased from Aladdin Biochemical Technology Co., Metronidazole (C10594013, Maclean), dicyclohexyl carbodiimide condensing agent (C11251817, AR99%), diethylamine, 4-dimethylamino pyridine catalyst (DMAP, AR99%, C11300958), absolute ethanol (AR99%), methanol (AR99%), dichloromethane (DCM, AR99%), concentrated hydrochloric acid (AR99%), glycerin was purchased from Maclean Biochemical Technology Co., Ltd. Amoxicillin (02-170404, Xiansheng pharmaceutical), Metronidazole (443-48-1, Macklin), levofloxacin (160680, Shandong Lukang Pharmaceutical Group Saite Co., Ltd.), Omeprazole (Jinan Mingxin Pharmaceutical Co., Ltd., 18031009), Brain Heart infusion (BHI, 293862), Columbia blood agar base (CBAB, 2938621) were purchased from the United Kingdom, Oxoid biological company, nutrient broth medium (Basebio, BS1003), nutrient agar (Basebio, BS1002), Sabouraud medium, soy peptone, beef extract, calf serum, Fetal bovine serum (Sangon, E600001-0500), PBS Buffer (Sangon, B040100-0005), RPMR medium1640 medium (KaiJi biology, KGM31800-500), Gram stain, bacterial genomic DNA extraction kit (Taingen, batch number: DP302), *Hp* 16sRNA specific primers, CCK8 kit (Beyotime, batch number: C0038); UV spectrophotometer 90 (UH5300), multiscan spectrum (Synergy H1), three-gas incubator (Huaxi YCP-100S), three-gas shaker, GES-1 (RRID: CVCL_EQ22), and BGC-823 (KeyGEN BioTECH, Nanjing, China) were used. (GES-1 and BGC-823 were provided by Professor Bi Hong kai of Nanjing Medical University).

### Strain and strain culture.

*Hp* strains: *Hp* standard strains 26695, G27, and Gastric mucosal colonization strain *Hp*159 in mice with multiple drug resistance to levofloxacin, clarithromycin, (all provided by Professor Bi Hongkai of Nanjing Medical University); 6 clinically Met-resistant strains (acquired by our laboratory from the gastric mucosae of clinical patients and identified as *Hp* through gram staining, urease test, oxidase and catalase, *CagA* gene detection, and DNA16sRNA sequencing); all *Hp* strains were stored in a brain heart infusion containing 30% glycerin at −80°C.

Non-*Hp* strains: Staphylococcus aureus, Escherichia coli, Pseudomonas aeruginosa, Acinetobacter baumannii, Klebsiella pneumoniae, Candida albicans, Cryptococcus neoformans, Saccharomyces cerevisiae, Bacillus subtilis, Stenotrophomonas maltophilia, Lactobacillus acidophilus, Acetobacter pasteurianus, Candida tropicalis, Lactobacillus Curvatus, Morganella morganii, Campylobacter jejuni, Bacteroides Fragilis, Bifidobacterium longum, Enterobacter hormaechei, Staphylococcus Haemolyticus (purchased from Guangdong Microorganism Conservation Center).

Before each experiment, the aforementioned strains were removed from the freezer where they were stored at −80°C, equilibrated to room temperature, and centrifuged to remove the preservation solution. After the remaining *Hp* were inoculated on a Columbia agar plate containing 10% calf serum, the precipitate was evenly coated with the inoculation loop and placed in a microaerophilic (10% CO_2_, 5% O_2_, 85% N_2_) at 37°C for 72 h, after which small colorless, transparent, small bacterial colonies were observed with the naked eye.

After two stable passages, an appropriate number of colonies were scraped from the agar plate, inoculated in a fresh brain heart infusion (BHI) medium containing 10% calf serum, and shaken at 170 rpm at 37°C under microaerobic conditions for 72 h. On the other hand, other non-*Hp* bacterias were cultivated in the broth medium and the special medium. Fungi were cultivated in the Sabouraud medium in an incubator at 37°C for 48 h, and allowed steady passage, they were then inoculated on the corresponding medium and cultured in an incubator at 37°C for 24 h.

### Methods.

**(i) Preparation of Lla-Met compound.** 1 mM linolenic acid, 5 mL dichloromethane, and DCC condensing agent that had been stirred at room temperature for a certain time, were dosed with 1 mM metronidazole, 5 mg DMAP, and 2 mL diethylamine, stirred overnight, after which a white precipitate was observed in the reaction solution. Thereafter, this solution was analyzed through thin-layer chromatography (TLC). After the linolenic acid was observed to have disappeared completely, the solution was filtered through diatomaceous earth. With the filtrate spin-dried through column chromatography, the target product Lla-Met was collected, which was a compound that was a yellowish oily substance and kept at −20°C away from light. Eventually, after Lla-Met was obtained, the carbon and hydrogen elements therein were detected by the magnetic resonance imaging, the mass spectrum, and the infrared spectroscopy, to infer its purity (its characterization was completed by Shanghai Nafu Biological Co., Ltd.).

**(ii) Detection of MIC of Lla-Met by microdilution.** Microdilution was used to detect the MIC of linolenic acid-metronidazole compound to standard *Hp* strains G27, *Hp* 26695 and clinically drug-resistant strains. With reference to the previous experimental method (24), corresponding adjustments were made. The specific steps were as follows: a serial 2-fold dilution of linolenic acid-metronidazole was prepared in a 96-microwell plate supplemented with fresh BHI medium that contained 10% serum. *Hp* in the logarithmic phase were diluted 10-fold in a cell suspension with OD_600 nm_ = 0.3 (about 1 × 10^8^ CFU/mL), which were inoculated on a 96-microwell plate, so that the final concentration of G27 was 1 × 10^6^ CFU/mL, and shaken at 170 rpm at 37°C under microaerobic conditions for 72 h. Metronidazole and linolenic acid were used as positive controls with PBS as a negative control. The MIC was the lowest drug concentration that inhibited 90% bacterial growth. Three biological repeats were performed for each experiment. However, other non-*Hp* strains were used to prepare a 96-microwell plate in the same way (the final working concentration of the bacterial solution was 1 × 10^5^ CFU/mL).

**(iii) Detection of MBC of Lla-Met to Met-resistant *Hp* through spread plate method.** Referenced to the previous experimental method (24). Serial dilutions (2×MIC, 4×MIC, 8×MIC, 16×MIC) of different concentrations of Lla-Met compound were prepared in the BHI medium supplemented with 10% serum in a 96-microwell plate. *Hp* 159 in the logarithmic phase was diluted and inoculated on a 96-microwell plate, with the final concentration of *Hp* brought to 1 × 10^6^ CFU/mL. Therefore, it was shaken at 37°C in a microaerobic shaker, with an equal volume of samples for each concentration removed at certain intervals (2 h, 4 h, 8 h) for serial dilution (1:1 to 1:1000), and the 100 μL diluted solution was coated evenly over the Columbia medium containing 10% serum and cultured at a microaerophilic incubator at 37°C for 72 h. Eventually the number of single bacterial colonies on the plate was counted, and MBC of Lla-Met was estimated by drawing a time-colony number (log 10) curve. MBC is the minimum drug concentration required to kill 99.9% of *Hp* (log 10^3^) within a certain time; PBS and metronidazole were used as controls. Three biological repeats were performed for each experiment.

**(iv) Construction of models of mice with acute gastritis and evaluation of therapeutic effects of Lla-Met *in vivo*.** SPF C57BL/6 mice were purchased from Changsha Tianqin Biological Co., Ltd., as approved by the Institutional Animal Care and Use Committee of You jiang Medical College and taken as experimental animals in accordance with animal welfare standards and institutional guidelines, under mice ethics review number: 2019112501. The tested *Hp* strain was Gastric mucosal colonization strain *Hp*159 in mice. The mice modeling was established specifically with reference to the experimental methods of Thamphiwatana, Xie et al. (25, 35), with appropriate adjustments made thereafter. The mice were made to fast for 12 h before gavage, BHI was used to prepare BHKS159 suspension and the concentration of bacterium was adjusted to OD_600 nm_ = 3 (1 × 10^9^) CFU/mL, which was then given to each mouse in a dose of 0.5 mL through oral-nasal feeding, then fasted and were deprived of water for 4 h. The gavage was given to the mice once every other day on five consecutive occasions. At the end, three mice were randomly sacrificed and their gastric tissue taken for identification; identification followed the protocol described above.

After the modeling was confirmed as having been established, the mice were randomly divided into four treatment groups: PBS, metronidazole + amoxicillin + Omeprazole (Met + AM + OPZ, standard triplet), linolenic acid-metronidazole compound + Omeprazole (OPZ + Lla-Met), and metronidazole + linolenic acid + Omeprazole group (Met + Lla + OPZ), with uninfected mice serving as negative controls. The dosage was 138.2 mg/kg omeprazole, 28.5 mg/kg amoxicillin, 30 mg/kg metronidazole + Linolenic acid and 24 mg/kg linolenic acid metronidazole compound. All treatment groups were given a Omeprazole (PPI) 30 min before the administration, after which they were deprived of food and water for 4 h and were administered treatment once a day for three consecutive days. 2 weeks after the last gavage, the mice were weighed, and the mean average body weight calculated. Then mice were fasting for 12 h, sacrificed through cervical dislocation and dissected to acquire stomach tissue. One part of the stomach tissue was stored in formalin while the other part was weighed, homogenized, diluted, coated and inoculated on the Columbia medium containing 10% serum. The medium was placed at a microaerophilic incubator at 37°C for 72 h, in which the colony morphology was observed and acicular transparent colonies were counted using the method described in Section 1.3.4 and identified through gram staining and 16S following the protocol described in Section 1.2.

**(v) Evaluation of Lla-Met toxicity *in vivo* and *in vitro*.** *(a) Evaluation of the cytotoxicity of Lla-Met compound in vitro.* The CCK8 assay was employed to evaluate the Cytotoxicity of Lla-Met on immortalized human gastric epithelial cell lines (GES-1) and human gastric cancer cells (BGC823). GES-1 and BGC823 cells were cultured in a 1640 medium containing 10% fetal bovine serum at 37°C in an incubator with 5% CO_2_. When cell confluence reached 90%, 1 × 10^5^ cells were laid on a 96-microwell plate and cultured overnight, with different concentrations of Lla-Met acting thereon for 24 h. These cells, however, were treated with CCK8 reagent, and finally detected by the multiscan spectrum at 450 nm to measure the absorbance, with each concentration of cells placed in three holes. Three biological repeats were performed for the each experiment. PBS was used as a negative control. Cell viability was expressed through cell inhibition rate. The formula for calculating cell inhibition rate is as follows: drug treatment group/PBS group × 100%.

*(b) Evaluation of the cytotoxicity of Lla-Met compound in vivo.* To evaluate the cytotoxicity of Lla-Met compound, the uninfected C57BL/6 mice were divided into two groups: the drug and PBS group. The administration group was given 10 times the treatment dose for three consecutive days, once a day. On the other hand, PBS was used as a control group, with the same dose and times of administration as the other group. The mice were weighed the day before administration of treatment and were weighed after administration for 7 days. Three days after withdrawal of treatment, mice in the administration group were weighed, subjected to cervical dislocation, with tissue taken from liver, spleen, and kidney for hematoxylin and eosin (HE) staining.

**(vi) Detection of *Hp’s* drug resistance to Lla-Met compound.** Specifically, with reference to the previous experimental method (24), G27 was exposed to Lla-Met compound at a subinhibitory concentration through serial passaging to evaluate the drug resistance of G27 to Lla-Met compound. The procedure was as follows: with the concentration in the logarithmic phase adjusted to 1 × 10^6^ CFU/mL in fresh BHI medium containing 10% serum, the G27 were allocated to a 24-well plate, treated with Lla-Met with the final concentration brought to 1/4 of Lla-Met MIC (0.25 μg/mL). Thereafter, the given cell suspension was shaken on a shaker for 3 days, after which the optical density value of the cells in each well was detected at OD_600 nm_ with a microplate reader. At the same time, the MIC of the cell culture was detected and recorded with the induction lasting for 18 days. When there were changes in MIC, the concentration of Lla-Met was adjusted over time. Metronidazole was used as a control.

**(vii) Stability testing of Lla-Met in an acidic environment.** *(a) Analysis of the stability of Lla-Met through thin-layer chromatography (TLC)*. Absolute ethyl alcohol was used to dissolve Lla-Met; solutions of HCl/KCl buffer (0.2 mM) at pH 3.0 and 7.0 were prepared and the above prepared Lla-Met was added to pH 3.0 and 7.0 and placed at room temperature. DCM and methanol were prepared in the ratio of 20:1 (vol/vol) and saturated at room temperature for 2 min. A horizontal line was lightly drawn 10 mm from the bottom of a TLC plate and a capillary dropper was employed to absorb Lla-Met solutions with pH 3.0 and 7.0 and dot them in different positions on the horizontal line. The TLC plate was placed in the developing tank and removed after the developing solvent advanced on the TLC plate and reached a certain distance. The spots formed by the same substances were found to be at the same level after being observed on the plate under a 254 nm UV lamp.

*(b) Analysis of the stability and bactericidal activity of Lla-Met through plate coating method*. Fresh BHI containing 10% serum was prepared into pH 3.0, pH 4.5 and pH 6.0 bacterial culture medium with sterile HCl/KCl (0.2 mM) and NaOH/KCl (0.2 mM) buffer solution. Thereafter, nutrient solutions were distributed across a 96-microwell plate. Meanwhile, serial dilutions of Lla-Met compound of different concentrations were prepared (2×MIC, 4×MIC) and the BHI was adopted to dilute the G27 bacteria in the logarithmic phase. The rest steps were referenced the above PBS and metronidazole were used as controls. Three biological repeats were performed for each experiment. MBC is the minimum drug concentration required to kill 99.9% of *Hp* (log 10^3^) within a certain time.

### Statistical analysis.

The statistical analysis was performed using the SPSS 17.0 statistical software. The measurement data were expressed as “mean ± standard deviation.” The comparison between two sample means was conducted by the *t* test; the comparison between multiple sample means was carried out by the single factor variance analysis, with *P < *0.05 considered to represent a statistically significant difference.
