# Supplementary material for: Linolenic Acid-Metronidazole: a Compound Relieving Drug Resistance and Inhibiting Helicobacter pylori
Source: Antimicrob Agents Chemother. 2022 Jun 27;66(7):e00073-22. doi: 10.1128/aac.00073-22 (PMC9295599; doi:10.1128/aac.00073-22)
Supplement: Supplemental file 1 — Fig. S1 to S4. Download aac.00073-22-s0001.pdf, PDF file, 0.2 MB [file aac.00073-22-s0001.pdf]

Supplementary information

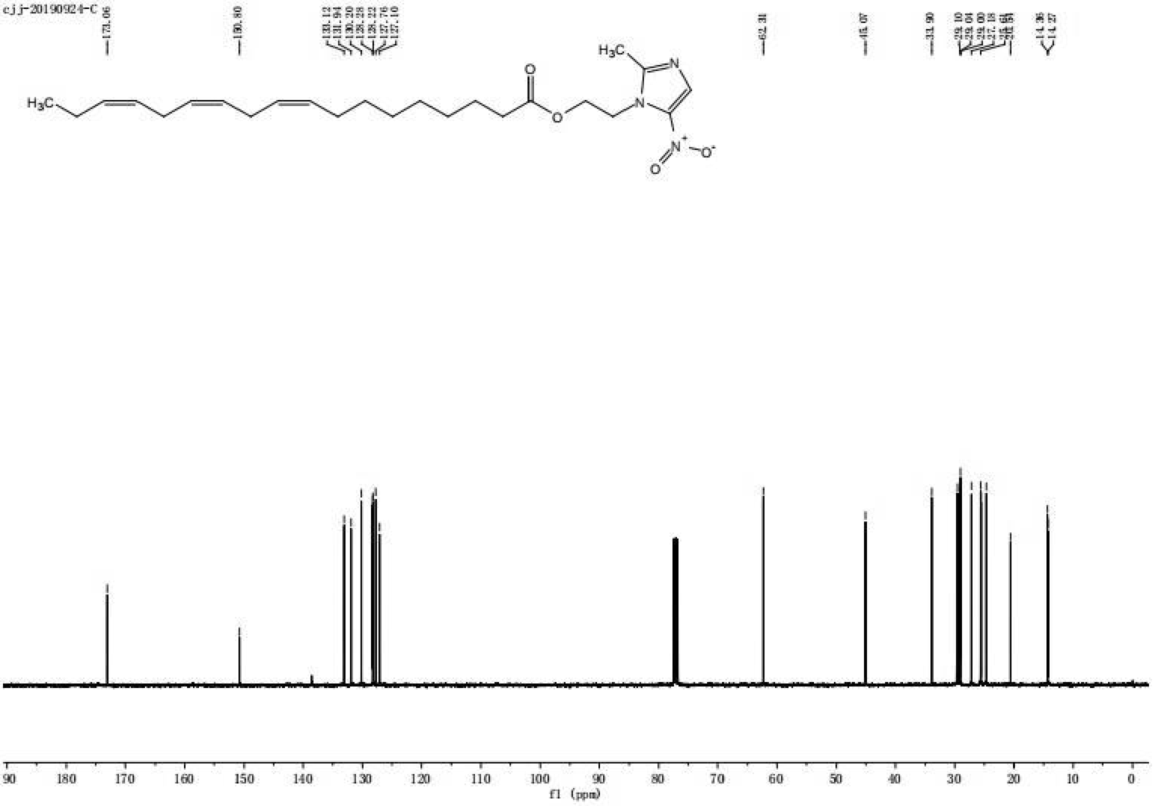

Supplementary Fig.1 Carbon spectrum of Lla-Met with nuclear magnetic resonance. [13] C NMR ($CDCl_3$ 100MHz) δ 173.06; 150.80; 133.12; 131.94; 130.20; 128.28; 128.22; 127.76;127.10; 62.31; 45.07; 33.90; 29.69; 29.54; 29.10; 29.04; 29.00; 27.18; 25.61; 25.52; 24.86; 20.54;14.36; 14.27.

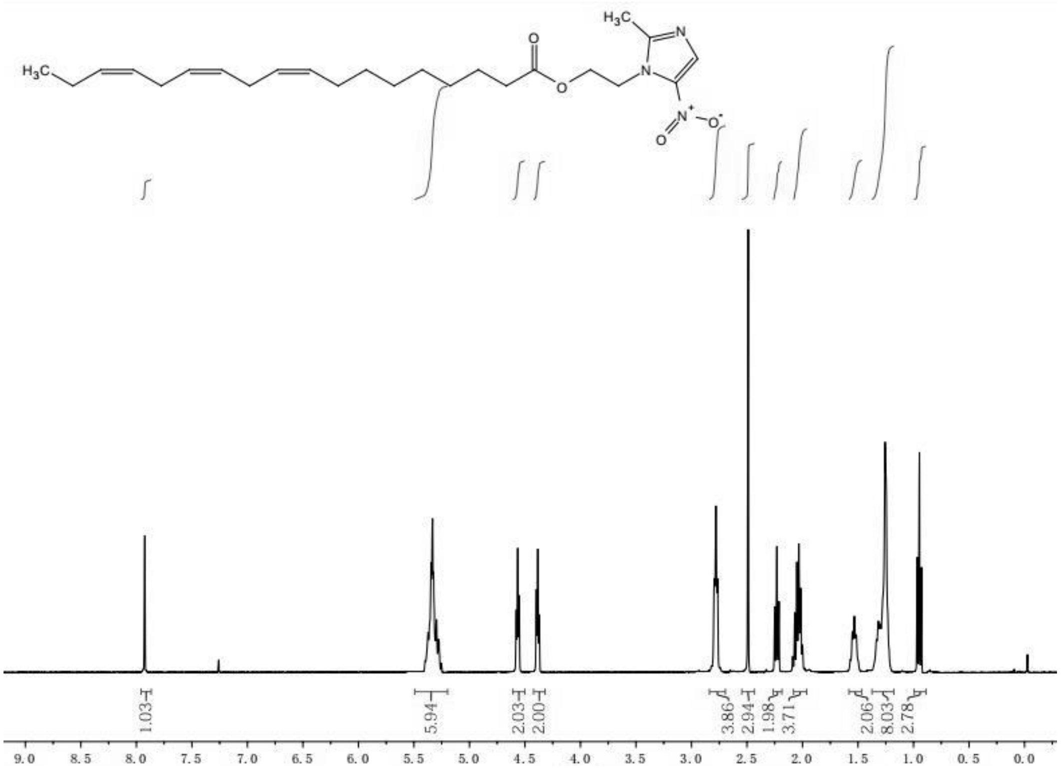

Supplementary Fig.2 Hydrogen spectrum of Lla-Met with nuclear magnetic resonance. ^{1}H NMR($CDCl_3$ 400MHz) δ 7.92 (s,1H); 5.24-5,41 (m, 6H); 4.56 (t, 2H, *J*=4.8 Hz); 4.38 (t, 2H, *J*=4.8Hz); 2.74-2.82 (m, 4H); 2.44 (s, 3H); 2.23 (t, 2H, *J*=7.2 Hz); 1.98-2.1 (m, 4H); 1.53-1.48 (m, 2H);1.20-1.36 (m, 8H)2z; 0.95 (t, 3H, *J*=7.6 Hz).

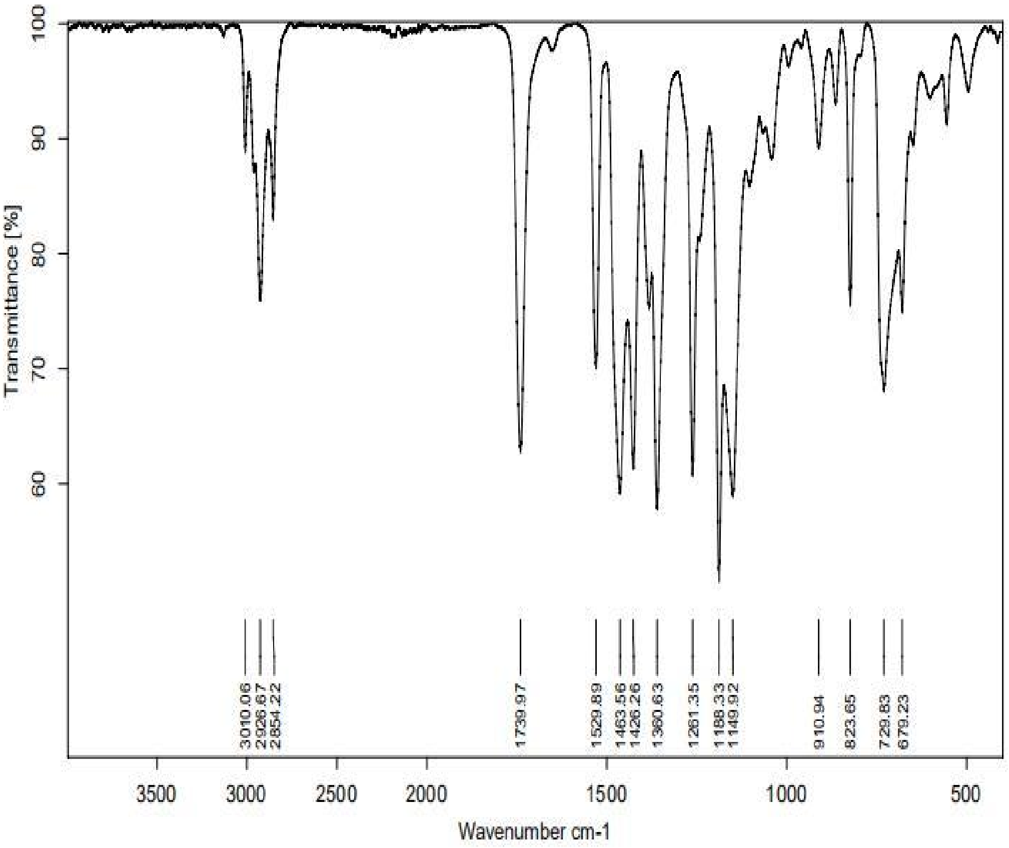

Supplementary Fig.3 Fourier infrared spectroscopy of Lla-Met. 3010cm$^{-1,}$ C-H expansion of C=CH; 2926 cm^{-1}, C-H symmetric stretching of alkyl groups; 2854 cm^{-1}, C-H antisymmetric stretching of alkyl groups; 1740 cm^{-1}, C = O stretching of ester; 1530 cm^{-1}, NO_2 antisymmetric expansion; 1361 cm^{-1}, NO2 symmetric expansion; 730 cm $^{-1}$, CIS HC = C-H out of plane bending of C-H.

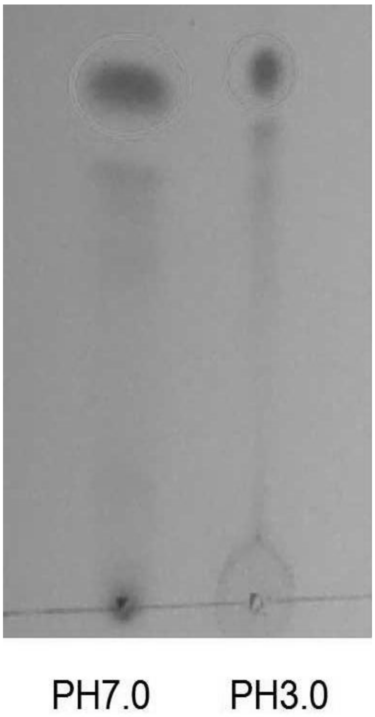

Supplementary Fig.4 Stability of Lla-Met with TCL analysis.
